# Classification of Fracture Risk in Fallers Using Dual‐Energy X‐Ray Absorptiometry (DXA) Images and Deep Learning‐Based Feature Extraction

**DOI:** 10.1002/jbm4.10828

**Published:** 2023-10-19

**Authors:** Damith Senanayake, Sachith Seneviratne, Mahdi Imani, Christel Harijanto, Myrla Sales, Peter Lee, Gustavo Duque, David C. Ackland

**Affiliations:** ^1^ Department of Biomedical Engineering University of Melbourne Parkville VIC Australia; ^2^ Department of Mechanical Engineering University of Melbourne Parkville VIC Australia; ^3^ Melbourne School of Design University of Melbourne Parkville VIC Australia; ^4^ Australian Institute for Musculoskeletal Science (AIMSS), Geroscience & Osteosarcopenia Research Program University of Melbourne and Western Health St Albans VIC Australia; ^5^ Department of Medicine‐Western Health Melbourne Medical School St Albans VIC Australia; ^6^ Bone, Muscle & Geroscience Group, Research Institute of the McGill University Health Centre Montreal QC Canada; ^7^ Dr. Joseph Kaufmann Chair in Geriatric Medicine, Department of Medicine McGill University Montreal QC Canada

**Keywords:** AGING, ANALYSIS/QUANTITATION OF BONE, BIOENGINEERING, DXA, FRACTURE RISK ASSESSMENT, ORTHOPAEDICS, PRACTICE/POLICYRELATED ISSUES

## Abstract

Dual‐energy X‐ray absorptiometry (DXA) scans are one of the most frequently used imaging techniques for calculating bone mineral density, yet calculating fracture risk using DXA image features is rarely performed. The objective of this study was to combine deep neural networks, together with DXA images and patient clinical information, to evaluate fracture risk in a cohort of adults with at least one known fall and age‐matched healthy controls. DXA images of the entire body as, well as isolated images of the hip, forearm, and spine (1488 total), were obtained from 478 fallers and 48 non‐faller controls. A modeling pipeline was developed for fracture risk prediction using the DXA images and clinical data. First, self‐supervised pretraining of feature extractors was performed using a small vision transformer (ViT‐S) and a convolutional neural network model (VGG‐16 and Resnet‐50). After pretraining, the feature extractors were then paired with a multilayer perceptron model, which was used for fracture risk classification. Classification was achieved with an average area under the receiver‐operating characteristic curve (AUROC) score of 74.3%. This study demonstrates ViT‐S as a promising neural network technique for fracture risk classification using DXA scans. The findings have future application as a fracture risk screening tool for older adults at risk of falls. © 2023 The Authors. *JBMR Plus* published by Wiley Periodicals LLC on behalf of American Society for Bone and Mineral Research.

## Graphical abstract

A modeling pipeline was developed for fracture risk prediction using DXA images. Small vision transformers are a neural network technique that show high quality fracture risk classification using DXA scans.

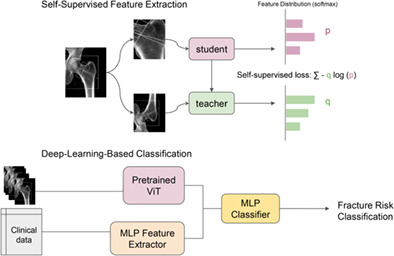

## Introduction

Fractures in older adults are common and often lead to long rehabilitation periods,^[^ ^1^ ^]^ reduced quality of life,^[^ ^2^ ^]^ and high cost to social and healthcare systems.^[^ ^3^, ^4^ ^]^ Falls are a strong predictor of imminent fractures in older men and women.^[^ ^5^ ^]^ They are the most frequent cause of unintentional injuries, particularly among older adults (aged ≥65 years), and are the leading cause of emergency admission, loss of functional ability, independence, quality of life, and injury‐related death.^[^ ^6^ ^]^ Identifying factors that may associate an individual with a higher risk of falls and fracture has important implications for preventative care, but has remained a long‐standing challenge.

DXA scans are one of the most commonly used imaging techniques for estimating bone mineral density (BMD),^[^ ^7^ ^]^ because scanning is typically low cost, with low radiation dose, and can be used to image the entire body at once. Although BMD data from DXA scans are used to identify patients with established osteoporosis, the assessment of fracture risk using these data cannot be discerned directly and depend on proprietary modeling or data analytics approaches.^[^ ^8^ ^]^ Other fracture risk calculation algorithms such as Fracture Risk Assessment Tool (FRAX)^[^ ^9^ ^]^ and Garvan^[^ ^10^ ^]^ typically combine factors such as patient demographics, past fracture history, and health status with BMD data (see^[^ ^11^ ^]^ for a review); however, these approaches do not explicitly consider region‐specific bone structure information, nor other biomarkers that may be associated with bone quality, including vitamin D and calcium levels.

Machine learning strategies, such as deep neural network models, have played a role in the identification of musculoskeletal conditions using images, including automated tumor classification from magnetic resonance imaging (MRI) and computed tomography (CT), detection of spinal fracture, and calculation of bone age and fragility.^[^ ^12^ ^]^ A significant challenge in developing these image processing methods has been the availability of large image datasets with relevant fracture risk labels, which are required for effective model training and fracture risk prediction. Ultimately, small image datasets may not sufficiently represent the distribution of previous fractures throughout the body to allow accurate and robust, automated fracture classification.

Augmentation of image datasets using approaches such as synthetic minority oversampling, which repeatedly resamples and combines images from an existing dataset, has been used to overcome the challenges of model training using low sample sizes^[^ ^13^ ^]^; however, such approaches assume that there exists a smooth, continuous distribution of labels, which may not be the case in reality and may ultimately result in model training using erroneous labels. For instance, the average of two images with one fracture in each may be an image with two fractures, though the label assigned would be representative of a single fracture. Self‐supervised learning has been recently proposed as a strategy for model training in the absence of large, labeled datasets and avoids these label distribution assumptions. An input dataset is used to train a neural network model without using label information to identify low‐level features of the data, such as edges, curves, and contours, which can make the final classification task more efficient.^[^ ^14^ ^]^ Self‐supervised learning pretraining has also been observed to improve downstream classification accuracy in the absence of a large, labeled dataset to extract image features from.^[^ ^15^ ^]^ This approach has shown promise in medical image classification of label sparse datasets, including chest X‐rays and skin dermatology images,^[^ ^16^ ^]^ but to date has not been applied in fracture risk classification using DXA images.

The objective of this study was to employ artificial neural networks, together with DXA images and patient clinical information, to evaluate fracture risk in a cohort of fallers and age‐matched healthy controls. This study proposes a unique approach to automatic fracture risk classification using bone structural information derived from DXA images.

## Subjects and Methods

### Study population

Data from 526 community‐dwelling older adults presenting to a Falls and Fracture Clinic in Melbourne, Australia, between October 2016 and January 2022 were used for cross‐sectional analysis. Inclusion criteria were: aged ≥65 years; able to mobilize independently or using a gait aid (walking stick, frame, etc.); no severe cognitive deficits (Mini‐Mental State Exam >18); and at least one risk factor for falls or fractures. This included 196 individuals with at least one fall (mean age: 77.3 ± 6.7 years, mean weight: 70.4 ± 15.7 kg), and 282 individuals with two or more falls (mean age: 77.8 ± 7.2 years, mean weight: 74.1 ± 12 kg). In addition, 48 age‐matched controls with no fall history were recruited (mean age: 77.6 ± 6.1 years, mean weight: 72.8 ± 18.6 kg). In this cohort, 111 patients had no fracture history, 329 patients had one fracture and 92 patients had two or more fractures. The study was approved by the Western Health Low‐Risk Ethics Panel at Sunshine Hospital (ID: QA2018.106_44499). Written informed consent was waived as data was collected as part of standard care.

### Falls and fracture definitions

Falls were defined as “unexpected and involuntary loss of balance, causing the person an undesired contact with the ground.”^[^ ^17^, ^18^, ^19^ ^]^ The occurrence of falls in participants was assessed retrospectively by asking each participant (i) whether they had suffered a fall and, (ii) the number of falls experienced in the year before the day of the assessment. In the present study, only historic osteoporotic fragility fractures occurring in the previous 5 years were part of the inclusion criteria, defined as low‐trauma such as the forces equivalent to a fall from standing height or less.^[^ ^20^ ^]^ The number of self‐reported fractures were documented and subsequently validated against medical records, including discharge summaries, radiology reports, and referral letters. Participants reporting fractures that could not be verified by medical records were excluded from the analysis. Fracture risk was subsequently categorized for each subject based on fracture history and included (i) low fracture risk—no prior fracture; (ii) moderate fracture risk—one past fracture; and (iii) high fracture risk—two or more previous fractures. This fracture risk categorization was considered independent to fall risk or the number of falls experienced by participants, which were not predicted in the present study.^[^ ^21^ ^]^

### Image acquisition and analysis (DXA)

BMD and body composition (fat and lean mass) were assessed using a Hologic Horizon DXA machine (Hologic Inc., Bedford, MA, USA). BMD of the hip and lumbar spine were estimated in array mode according to the manufacturer's protocols and software. Using the DXA machine custom analysis software, standard landmarks (scapulohumeral joint space and femoral neck) were identified on whole‐body scans, and limbs were separated from the corpus and pelvis. Appendicular lean mass (ALM) was subsequently calculated for each region. Daily and monthly calibration of the DXA machine for BMD, muscle and fat masses were carried out using the spine and whole‐body phantoms. A single experienced image analysis specialist carried out all imaging and image analyses. DXA images of the entire body, as well as isolated images of the hip, forearm, and spine, were obtained from all faller and non‐faller subjects. This included 1488 images, which excluded instances where patients could not assume the required positions or where images were excessively noisy. Images were exported in Digital Imaging and Communications in Medicine (DICOM) format, manually cropped to include only relevant anatomical information, and resized to a 224 × 224 pixel image for subsequent modeling.

### Artificial neural network development

Artificial neural networks were developed to classify patient DXA images with and without tabular clinical data into the three fracture‐risk categories. To achieve this, images from the hips of subjects across the entire image dataset were first randomized then split into train, validate, and test image sets comprising 80%, 10%, and 10% of the images, respectively. During the training, validation accuracies were obtained after each training epoch and the model saved at the epoch with the highest validation accuracy. The results reported were calculated based on predictions provided by that model when the hold‐out test image set was provided as input.

**Fig. 1:**
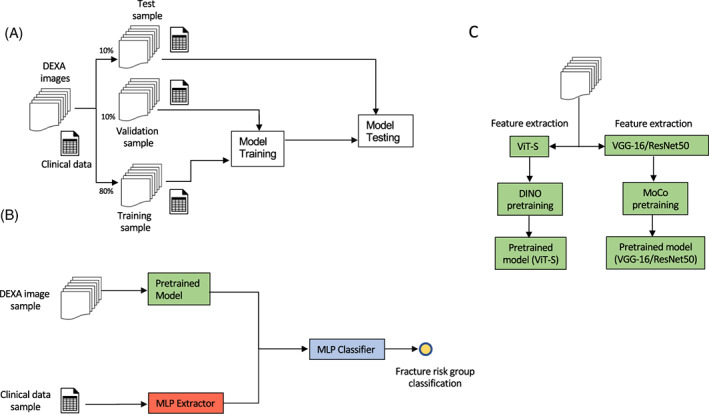
Artificial neural network modeling and validation pipeline for fracture risk prediction (*A*), inner model architecture allowing for image and non‐image data to be combined into fracture risk group classifications (*B*), and the two strategies used for pretraining feature extractors using self‐supervised learning (*C*). Each dataset derived from the complete DXA image superset of fracture patients and healthy controls were spit into a training sample (80%), validation sample (10%) and a test sample (10%). The model pretraining feature extraction strategies employed were Small Vision Transformer (ViT‐S) and Distillation with No Labels (DINO), while classification was performed using Convolutional Neural Network Models (VGG‐16 and ResNet‐50) and Momentum Contrastive Learning (MoCo). After pretraining, the feature extractors of the trained ViT‐S and VGG‐16/ResNet‐50 neural networks were then paired with a multi‐layer perceptron (MLP), which was used for fracture risk classification. Tabular clinical data were also included in the classifier using an MLP extractor.

Two types of artificial neural network models were employed for fracture risk classification: (i) Convolutional Neural Networks (ConvNets) and (ii) small vision transformer networks (ViT‐S) (Fig. 1A–C). ConvNets utilize the pixel geometry of images to extract features by sliding a learnable template along the two axes of the images. ViT‐S, an emerging technique that rivals ConvNets in power to extract image features, uses a more general feature extractor than the template‐matching approach of ConvNets called multihead self‐attention. Building on sequence data processing methods commonly used in language processing (NLP), ViT‐S models adopt a linear transformation instead of deep convolutional layers and leverage the two‐dimensional sequential nature of images to extract image features (see Supplementary Material).

To improve model classification accuracy given the relatively low data sample sizes, two different self‐supervised pretraining approaches were employed.^[^ ^22^, ^23^ ^]^ Specifically, Momentum Contrastive Learning (MoCo) and Distillation with No Labels (DINO) (Fig. 1). To represent ConvNets, two widely used architectures were adopted: VGG‐16 and ResNet‐50 model architectures (see Supplementary Material for details). Transfer learning and pretraining involves the use of a large unlabeled dataset for self‐supervised feature‐extractor learning. Our pretrained feature‐extractor used the full set of available DXA images from all modalities without label information specifying fracture history. Once the feature‐extractor was initially pretrained with self‐supervised learning, a classifier head made up of a Multilayer Perceptron (MLP) network was used to complete the classification model. Fine‐tuning of the MLP classification head was performed using the fracture risk labels associated with only the hip images, because previous studies have employed BMD of the femoral neck in calculations of fracture risk.^[^ ^24^ ^]^ For both MoCo and DINO, standard hyperparameters recommended in the software libraries were employed (see Table S1, Supplementary Material for further information).

#### Integration of clinical data

The effectiveness of using subject‐specific clinical data for fracture risk prediction was also evaluated, and this included subject vitamin‐D level,^[^ ^25^ ^]^ calcium level,^[^ ^26^ ^]^ height,^[^ ^27^ ^]^ and the ratio of appendicular lean mass to body mass index (ALM/BMI)^[^ ^28^ ^]^ (see Supplementary Material for calculation of vitamin D and calcium levels). These variables were included because of their known association with fracture likelihood (see Supplementary Material). DXA‐derived information such as BMD and *T* values for the hip and femoral neck were excluded, because relevant bone structural information was derived directly from DXA images using the neutral networks. The clinical data were fed into a MLP model with one hidden layer, and fracture risk classification was repeated.

To evaluate change in model performance with the integration of both DXA images and clinical data, the highest performing model trained only on imaging data was selected and used for subsequent analysis. Model performance was achieved by passing the clinical data through a trainable MLP in order to learn the optimal feature combinations. The output of the MLP with the image latent features were obtained through the feature extraction backbone. The concatenated features were then passed through the second MLP, which acted as a classification head (Fig. 1B). Although the image feature extraction back‐end was frozen to retain the latent features learned through pretraining, both MLP models were trained through back‐propagation. By employing the first MLP model, we effectively increased the network depth trained on the clinical data without loss of information from the extracted image features using the second MLP head.

### Artificial neural network model evaluation and validation

To benchmark the performance of the proposed neural network workflows, we used the VGG‐16 model pretrained on the Imagenet [VGG‐16 (Imagenet)] natural image dataset as the baseline fracture classification model (model 1), because this modeling approach is most commonly used in medical image analysis. Our pilot study also showed that VGG‐16 generally outperformed its later incarnation, VGG‐19, possibly owing to the smaller number of parameters in the VGG‐16 model, which may be an advantage with small datasets. This baseline model was then compared to the VGG‐16 model pretrained on the DXA images using the MoCo strategy [VGG‐16 (MoCo)] (model 2), Resnet‐50 model with the MoCo pretraining [Resnet‐50(MoCo)] (model 3), the Vision Transformer model pretrained with the DINO strategy [ViT (DINO)] (model 4), the use of clinical data only (model 5), and the combination of clinical data with the best‐performing image processing strategy [ViT (DINO) + clinical data] (model 6). In all cases where pretraining was used, random initializations of the feature‐extractor was performed. Class‐specific reweighting adapted from Seneviratne et al.^[^ ^29^ ^]^ was used to overcome the class‐imbalances between the three classes (see Supplementary Material for equation). The self‐supervised pretraining helped to mitigate overfitting following reweighting. The accuracy of each neural network's fracture‐risk group predictions was measured using the F1 Score with microaveraging (F1 Micro) and the area under the receiver‐operating characteristic curve (ROC‐AUC) score.

To validate the model performance and sensitivity to random sampling, we conducted a 10‐fold cross‐validation on the highest performing model. This cross‐validation was performed using only the highest‐performing model in order to reduce neural network training time. To achieve this, we split the complete dataset into two random groups of data, a train‐validate set comprising 90% of the images, and holdout test set with the remaining 10% of the images. The train‐validate set was then further split into validation and training sets by randomly sampling images such that 10% of the full image set was in the validation set and 90% were in the training set. This train‐validate split process was repeated nine times, at each time training a new model with the training set and selecting the best model iteration with the validation test. Once trained, each of the models were tested on the held‐out test set.

## Results

The accuracy of classifying DXA image data into fracture‐risk groups using the baseline model [VGG‐16 (Imagenet)] (model 1) was 66.7% (F1‐Micro) and 69.1% (ROC‐AUC) (Table 1). With class‐specific reweighting, the ROC‐AUC was reduced to 64.8% with no change to the F1 scores. The VGG‐16 (MoCo) model (model 2) had similar F1 scores, and the unbalanced and rebalanced scenarios resulted in ROC‐AUC scores of 58.8% and 47.1%, respectively. In contrast, Resnet‐50 (MoCo) (model 3) had a significant change when class rebalancing was applied, with the ROC‐AUC scores increasing from 60.7% to 72.0%, and the F1 Score increasing from 63.6% to 66.7%. In model 3, we also observed that the class‐specific scores show less trivial predictions when rebalancing is applied i.e., the capacity to predict all images in a single class.

**Table 1:** Fracture risk group classification performance for artificial neural network models used in the present study, including F1 and area under the receiver‐operating characteristics curve (in parentheses)

	Balanced				Unbalanced			
	Low risk	Moderate risk	High risk	Average	Low risk	Moderate risk	High risk	Average
Clinical data only	0.0 (61.1)	80.0 (66.9)	0.0 (66.4)	66.7 (64.8)	0.0 (61.1)	80.0 (66.9)	0.0 (67.1)	66.7 (65.1)
VGG16 (Imagenet)	0.0 (62.3)	80.0 (69.4)	0.0 (62.9)	66.7 (64.9)	0.0 (69.1)	80.0 (66.1)	0.0 (72.1)	66.7 (69.1)
VGG16 (MoCo)	0.0 (56.8)	80.0 (46.7)	0.0 (37.9)	66.7 (47.1)	0.0 (60.5)	80.0 (74.4)	0.0 (41.4)	66.7 (58.8)
Resnet 50 (MoCo)	25.0 (66.7)	79.2 (69.4)	0.0 (80.0)	66.7 (72.0)	0.0 (56.2)	77.8 (68.0)	0.0 (57.9)	63.6 (60.7)
Resnet 50 (DINO)	0.0 (48.8)	15.4 (42.1)	29.4 (37.9)	21.2 (42.9)	0.0 (63.0)	0.0 (56.2)	26.3 (86.4)	15.2 (68.5)
ViT‐S (MoCo)	0.0 (71.6)	80.0 (58.7)	0.0 (52.9)	66.7 (61.0)	0.0 (67.9)	80.0 (62.8)	0.0 (57.1)	66.7 (62.6)
ViT‐S (DINO)	33.3 (68.5)	57.9 (69.0)	20.0 (78.6)	45.5 (72.0)	15.4 (54.3)	69.6 (61.6)	28.6 (71.4)	54.5 (62.4)
ViT‐S (DINO) + Clinical data	37.5 (67.9)	80.0 (72.7)	0.0 (82.1)	63.6 (74.3)	25.0 (66.7)	75.6 (72.3)	0.0 (86.4)	57.6 (75.1)

*Note*: Scores for each model were calculated using test datasets on the basis of that model's ability to predict fracture risk as either high, moderate or low risk. Class‐specific fracture risk classification calculated for each model configuration are listed as Low Risk, Moderate Risk, and High‐Risk, and average scores across classes are provided.

In contrast, ViT‐S + DINO (model 4) had an unbalanced classification performance of 54.5% (F1) and 62.4% (ROC‐AUC). With rebalancing, this classification performance became 45.5% and 72.0% for F1 and ROC‐AUC, respectively. This score is comparable with the performance of model 3; however, from the class‐specific scores, it was observed that ViT‐S + DINO predictions were less trivial. Using only the clinical data for classification (model 5), a fracture risk classification performance of 66.7% (F1) and 65.1% (ROC‐AUC) was obtained. The integration of both DXA images and clinical data (ViT‐S + DINO, model 6) yielded a performance of 57.6% (F1) and 75.1% (ROC‐AUC) for the unbalanced case, and 63.6% (F1) and 74.3% (ROC‐AUC) for the class‐weighted case, demonstrating an increase of F1 score with no significant drop in the ROC‐AUC scores. Although this was the highest performing neural network model, the class‐specific F1 scores showed that the introduction of the clinical variables increased the triviality of the predictions through 0% F1 scores for the high‐risk category.

The k‐fold cross‐validation results of the ViT‐S + DINO model when using DXA images and clinical data demonstrated a mean F1 score of 62.6% (standard deviation: 3.2%) and mean ROC‐AUC score of 74.2% (standard deviation: 3.0%).

## Discussion

The present study showed that artificial neural networks were able to correctly categorize fracture risk using DXA scans with a ROC‐AUC accuracy of up to 74.3% (Table 1). This high‐performance neural network was achieved using the ViT‐S model and the DINO pretraining strategy, combining both DXA images with tabulated patient clinical data. This finding demonstrates the utility and importance of combining different data sources and formats in classification of fracture risk. Although F1 score was mostly stable throughout all experiments, this may be a result of the class imbalances which the class‐reweighting strategy mitigated, as demonstrated by an increase in ROC‐AUC score. Specifically, the ResNet + MoCo scenario showed a classification performance improvement of ~12% with the class re‐weighting strategy, underscoring the importance of class rebalancing in highly unbalanced datasets. Despite improvement in ROC‐AUC scores, for most of the configurations the predictions were trivial, like due to a label imbalance. However, with pretraining using both ResNet + MoCo and ViT‐S + DINO, the class‐specific F1 Scores resulted in less trivial predictions.

Although CNNs have a built‐in bias (i.e., the localness and translational invariance of image features), transformers have a more general bias making them more difficult to train with smaller datasets. As such, transformers may be better equipped to identify complex features in data when provided with sufficiently large datasets. In the case where such large datasets are not available, transformers have been shown to improve model performance when pretraining is employed.^[^ ^30^ ^]^ The results in the present study are consistent with this, showing that even without the inductive bias of convolutional neural networks, pretraining may provide a means to extract useful image features from small or label‐sparse datasets.

This study also showed that integration of clinical information to the deep‐learning pipeline improved the model performance when compared to use of DXA images alone. The stability of the models investigated in this study was demonstrated by the high mean value demonstrated in the k‐fold cross‐validation performed on the most performant model, and the relatively low standard deviations.

Overfitting is commonly encountered in artificial neural networks where the label distributions are imbalanced. However, we observed that in the case where pretraining techniques and ViT‐S are used, the overfitting issue is alleviated to a large extent. This is demonstrated by the ViT‐S (DINO) configuration having the highest non‐trivial prediction results in terms of the class‐specific F1 and ROC‐AUC scores, i.e., this model did not simply output the label of the most abundant class when class imbalances were present.

**Fig. 2:**
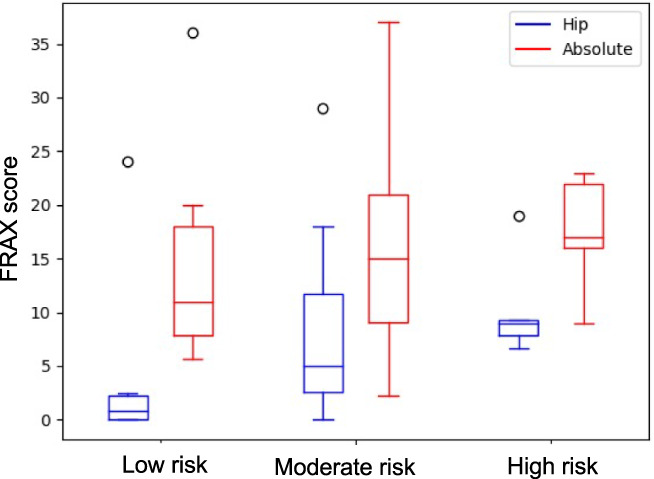
FRAX data representing hip fracture risk (blue) and absolute risk of major osteoporotic fractures (red).

Classification of fracture risk is clinically relevant, since accurate prediction of fracture events could trigger prompt preventive strategies, which have demonstrated to be effective in reducing the number of events and their devastating consequences.^[^ ^31^ ^]^ This is particularly relevant in older fallers at higher risk of bone fracture who may stand to benefit from tailored intervention. However, classification of fracture risk is challenging in clinical practice because some of the most commonly used fracture risk algorithms do not include falls history. In this study, we formulated our fracture risk‐groups based on fracture history. This risk score differs from FRAX, which also includes past fracture history, as well as other clinical and demographic data, and represents a 10‐year probability of a major osteoporotic fracture. Nonetheless, the fracture risk groups employed in this study show a similar increasing trend in fracture risk from low‐risk to high‐risk categories (Fig. 2), indicating that fracture history is a strong determinate of fracture risk.

The results of this study show that although ConvNets have a built‐in inductive bias catering for image recognition tasks, the ViT‐S models showed better overall improvement of performance compared to ConvNets. ConvNets produced more stable results, possibly due to built‐in inductive bias, which eliminates data hunger to an extent. However, the ViT‐S feature‐extractors performed considerably better when class‐imbalances are mitigated. This underscores the application of ViT‐S in settings where there are considerable class imbalances, together with a simple reweighting to improve gradient propagation.

**Fig. 3:**
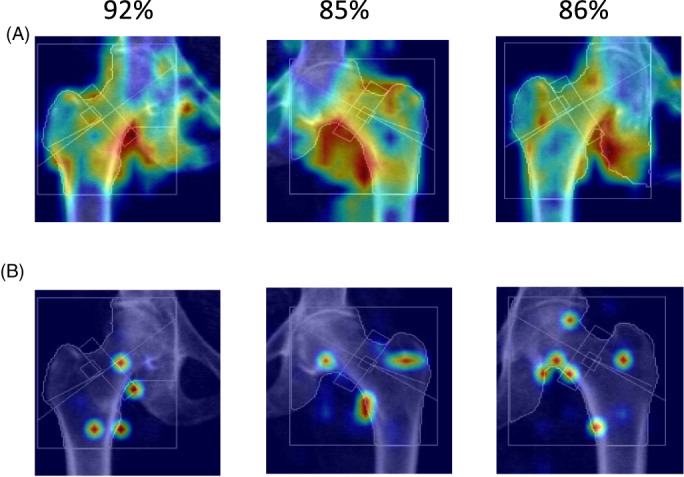
Visualization of EigenGradCAM (*A*) and GradCAM results (*B*) illustrating association between fracture group classification and DXA image features for moderate fracture risk subjects. Given are the three highest confidence images with labels predicted for correct classifications, which ranged from 85% to 92% confidence.

As an area for further research beyond this study, the capacity for neural networks to identify region‐specific (anatomical) areas associated with fracture on the DXA scans can be visualized using gradient‐based class activation maps (CAMs) (see Supplementary Material for details). In a preliminary exploratory study, we assessed EigenGradCAM (Fig. 3A) and EigenCAM (Fig. 3B) CAMs and showed that the femoral neck had high relevance in classifying moderate fracture risk. This may be due to the hip being a frequent fracture site in older adults, and the isolated hip DXA images having comparatively higher resolution than whole‐body DXA images and thus being most strongly associated with fracture risk.

We also observed from the CAM results that, for moderate fracture risk prediction, the inferred regions of interest generally resided within the bony anatomy visible on the DXA images. This may be due to the higher capacity of ViT‐S models for foreground extraction, and the model treating bony regions of the DXA images as foreground. It is also likely that the neural network model performance was improved by learned focused on signs of previous fractures present in the bones such as fracture lines, and presence of metal implants. In reality, fracture risk may be affected by more nuanced features such as the mechanical properties of the bones (length/cross‐section area), muscle architecture, and appendicular lean mass of subjects, which cannot be captured from DXA images alone. In future, it would be beneficial to extract both foreground and background segmentations through self‐supervised learning to be incorporated into the classification tasks. The use of a larger, higher resolution image dataset, or alternative imaging modalities for soft tissue to supplement the DXA scans, may also further improve classification accuracy.

Integration of tabular patient clinical data to the learning pipeline produced a considerable improvement in class imbalance mitigation. However, the performance did not improve significantly when compared to the improvements gained from the simple reweighting techniques. This suggest that it may be useful to include relevant clinical information in predictive models of fracture risk. Future research ought to focus on identifying the most relevant patient clinical variables to model when investigating the effectiveness of data preprocessing techniques to achieve improved classification performance.

There are limitations of the present study that ought to be considered. The DXA scans were of low resolution (390 × 261 pixels on average), making the self‐supervised learning of image features challenging. The small sample size of the dataset also may explain the limited improvements observed when clinical data were included, because the neural network parameters may require more data to be trained when more input variables are presented. In addition, the analysis was performed using two‐dimensional images, and further improvements in classification performance may be achieved by incorporating use of three‐dimensional modeling techniques for fracture prediction, such as finite element modeling.^[^ ^32^ ^]^ Finally, we performed our analysis on one complete dataset, which was validated using an internal hold‐out dataset and k‐fold cross‐validation, thus mitigating model overfitting. Nonetheless, future studies ought to assess model predictive capacity using a variety of different data sources to further validation of classification performance.

This study shows that artificial neural networks, together with DXA images and patient clinical data in fallers, can be used to classify fracture risk with high levels of accuracy beyond that of the DXA images or clinical data alone. In future research, larger high‐resolution image datasets may further improve fracture risk assessment and provide scope for gradient‐based class activation maps for identifying image‐based regions of interest on DXA scans that are indicative of fracture risk.

## Author Contributions

**Damith Senanayake:** Formal analysis; methodology; visualization; writing – original draft. **Sachith Seneviratne:** Formal analysis; methodology. **Mahdi Imani:** Data curation. **Christel Harijanto:** Data curation. **Myrla Sales:** Data curation. **Peter VS Lee:** Supervision. **Gustavo Duque:** Conceptualization; methodology; resources; writing – review and editing. **David C. Ackland:** supervision, conceptualization, methodology, resources.

## Funding information

Australian Research Council (LE170100200, FT200100098).

## Disclosures

The authors declare no conflicts of interest.

### Peer Review

The peer review history for this article is available at https://www.webofscience.com/api/gateway/wos/peer‐review/10.1002/jbm4.10828.

## Supporting information


Supplementary Material S1.
Table S1.

## Data Availability

No data are available.
