# Supplementary material for: Classification of Fracture Risk in Fallers Using Dual‐Energy X‐Ray Absorptiometry (DXA) Images and Deep Learning‐Based Feature Extraction
Source: JBMR Plus. 2023 Oct 19;7(12):e10828. doi: 10.1002/jbm4.10828 (PMC10731096; doi:10.1002/jbm4.10828)
Supplement: Supplementary file 1 — Supplementary Material S1. Table S1. [file JBM4-7-e10828-s001.docx]

**SUPPLEMENTARY MATERIAL**

*Clinical parameter selection*

An initial test was conducted to assess the relevance of specific clinical features in classifying bone fracture risk. This was achieved using feature importance tests provided in the scikit-learn Python library, i.e. the *mutual information test for classification*. However, the mutual information tests were affected by class imbalance, which could not be resolved using class-reweighting. We then performed a literature search to identify a set of features associated with fracture risk, which included Vitamin D and Calcium levels, subject height, the ratio of appendicular lean mass to body mass index (ALM/BMI), and femoral neck BMD [1-4]. After omitting femoral neck BMD, since the model was trained to obtain all relevant bone structural information directly from the DXA images using the neural networks, the final set of clinical variables used were Vitamin-D, calcium, height and ALM/BMI.

*Convolutional neural networks*

Convolutional Neural Networks were specifically designed for image processing tasks and utilise the two dimensionality of images. This approach considers the localness of the features (i.e. an edge or a patch of color is identifiable within a small neighborhood of pixels), and the translational invariance (i.e., a feature such as an edge or a patch of color may be present in multiple places in the image equivalent to a translation). Typically, these features are small, spanning only 3-7 pixels in width or height. Combinations of features are obtained by stacking layers of convolutional networks on top of each other. The field of vision is also adjusted for each feature by summarising the features through pooling operations or dilation operations. Since these networks are typically very deep, i.e. a large number of layers are stacked on top of each other, they are highly parametrised, and require large volumes of data to train. Furthermore, trainability issues also motivate introducing novel strategies of the architectural changes to accommodate stacking large numbers of layers [5].

*Vision transformers*

Vision Transformers are based on the revolutionary transformer architecture made popular in Natural Language Processing (NLP) [6], among other applications where learning from sequential data is required. By modelling an image as a 2-D sequence of patches and by using a positional embedding to encode the relative of patches with respect to each other, vision transformers are known to capture the global structure of images more effectively than convolutions, which suffer from a relatively limited receptive field.

Instead of built-in inductive biases capturing the specific geometries of the input-space, Transformers rely on implicitly capturing the inter-variable relationships of the input signals. This is achieved by employing self-attention of linear transformation of the inputs, along with using multiple attention heads to support multiple nonlinear features to be extracted. Extending this paradigm into computer vision, Vision Transformers (ViT) were proposed, using self-attention of linear transformations to replace Convolutional Layers. The caveat however, is that the Vision Transformers are data hungry. Recently, self-distillation with no labels (DINO) was proposed as a complement to the Vision Transformers, wherein self-supervised learning was employed to reduce the data-hunger in training ViT models.

*Hyperparameter Selection*

The training configurations used in the present study were achieved using a basic hyper-parameter tuning on the learning rate used in the stochastic gradient descent optimizers (Table S1).

Table 1: Hyperparameter settings used in the study. In most cases, best performance was achieved using the recommended hyperparameters in the relevant software libraries.

| Configuration | Hyperparameter Values | Reasons for inclusion |
| --- | --- | --- |
| DINO Pretraining (ViT-S, Resnet 50) | LR = 0.0005 | Default values for Imagenet |
| Fine-tuning DINO + Classifier | LR = 0.001 | Default values for Imagenet (testing on 0.0001, 0.0005, 0.01 different settings yielded lower performance/Failed to converge the loss) |
| Moco Pretraining | LR = 0.00001 | Larger values failed to converge the loss (0.0001, 0.001) |
| MoCo Fine-Tuning | LR = 0.0000001 | Larger values failed to converge the loss (0.00001, 0.0001) |

*Class-specific Rebalancing of Loss Function*

In the neural network training, categorical cross-entropy loss was used with specific class-weights for the three classes. For cross-validation settings, the class-specific weights change, however they were calculated with a single formula in all cases where the weight assigned to a class (*w_c_* ) was calculated using the following formula.

$$w_{c}=\frac{1-\beta}{1-\beta^{n_{c}}}$$

where , $\beta=0.999$, and $n_{c}=$ number of images in the class *c.*

*Pathology Parameters*

Venous blood was collected from resting subjects for the measurement of serum 25(OH) vitamin D3 (VitD), calcium, and albumin. Serum VitD concentration was measured by chemiluminescence using the Elecsys 25(OH)D3 assay (Roche). The intra- and interassay precisions were respectively 7.5 % and 10.6 %, (normal range=10-132 nmol/l). Serum calcium, albumin and creatinine were determined using automated standard laboratory methods. Because of the high prevalence of hypoalbuminemia in older adults, the serum concentration of albumin and calcium were used to correct the calcium value (calcium corrected value = Ca + 0.8 [40-albumin]). The calcium corrected value was used in the subsequent analysis. The clearance of creatinine was calculated from the Cockcroft formula ([(140-age in years) x weight (Kg)/ 72 x creatinine mol/l]). All measurements were performed at the pathology networks affiliated with Western Health (Melbourne, Australia).

*Model pretraining*

The MoCo pre-training approach generated multiple views of the same image through different augmentations, such as jitter, cropping and blurring [7]. The neural network was then pretrained on the task of correctly classifying pairs of augmented views of the same image without being confused by views originating from other images [8]. In contrast, DINO, a form of image categorisation, was used to learn fine-scale information from the image data [9]. The DINO pretraining approach generated multiple views from each image and mapped them onto a probability distribution across a large number of pseudo-categories. The consistency of this distribution between image views from the same image was then used as the basis for the training objective.

*Model architectures*

To represent ConvNets, two widely used architectures were employed, specifically, VGG-16 and ResNet-50 model architectures. The MoCo algorithm was applied to each of these two models with the training data, excluding the label information, to improve the feature-extraction capabilities of the convolutional layers of these models. In the classification task, the feature-extractors were coupled with a Multilayer Perceptron (MLP) head. The full architecture containing the feature extractor and the MLP head was then trained on the training set with the fracture risk category information provided as the label. A similar approach was adopted for ViT-S; however, the DINO algorithm was used to pretrain the feature-extractor. Since the tabular clinical data have different scales and means, and the scale and mean of the latent feature-spaces provided by the pretrained feature-extractors is variable, we used an MLP feature extractor to process these tabular variables into a latent representation. This facilitated concatenation of the two sets of latent features (derived from image and non-image data) to be analysed as input to the classifier MLP head.

*Regions of interest associated with fracture risk*

To visualize the regions of interest most strongly associated with fracture risk on DEXA scans, gradient-based class activation maps were employed [10]. Since different implementations of Class Activation Map (CAM) methods focus on different sized regions of interest, we considered two contrasting methods of class activation map visualizations to identify regions of interest contributing to class assignment: (i) EigenCAM and EigenGradCAM. Both methods were implemented using the pytorch-grad-cam library. The gradients of the last 4 transformer blocks of the ViT model for a given image were used for generating the CAM image, as these were the blocks that were directly connected via a fully connected linear layer to the predictive output. The calculated gradients, which are indicative of the influence of the respective features to the final output of the model, were then used to color grade the input images, resulting in region of interest shadings.

*Vitamin-D and calcium measurements*

Venous blood was collected from resting subjects for the measurement of serum 25(OH) vitamin D3 (VitD), calcium, and albumin. Serum VitD concentration was measured by chemiluminescence using the Elecsys 25(OH)D3 assay (Roche). The intra- and interassay precisions were respectively 7.5 % and 10.6 %, (normal range=10-132 nmol/l). Serum calcium, albumin and creatinine were determined using automated standard laboratory methods. Because of the high prevalence of hypoalbuminemia in older adults, the serum concentration of albumin and calcium were used to correct the calcium value (calcium corrected value = Ca + 0.8 [40-albumin]). The calcium corrected value was used in the subsequent analysis. The clearance of creatinine was calculated from the Cockcroft formula ([(140-age in years) x weight (Kg)/ 72 x creatinine mol/l]). All measurements were performed at the pathology networks affiliated with Western Health (Melbourne, Australia).

**REFERENCES**

[1] Wang N, Chen Y, Ji J, Chang J, Yu S, Yu B. The relationship between serum vitamin D and fracture risk in the elderly: a meta-analysis. J Orthop Surg Res. 2020;15:81.

[2] Li PF, Lin ZL, Pang ZH, Zeng YR. Does serum calcium relate to different types of hip fracture? A retrospective study. Chin J Traumatol. 2016;19:275-7.

[3] Armstrong ME, Kirichek O, Cairns BJ, Green J, Reeves GK, Valerie Beral for the Million Women Study C. Relationship of Height to Site-Specific Fracture Risk in Postmenopausal Women. J Bone Miner Res. 2016;31:725-31.

[4] Harvey NC, Kanis JA, Liu E, Cooper C, Lorentzon M, Bea JW, et al. Predictive Value of DXA Appendicular Lean Mass for Incident Fractures, Falls, and Mortality, Independent of Prior Falls, FRAX, and BMD: Findings from the Women's Health Initiative (WHI). J Bone Miner Res. 2021;36:654-61.

[5] He K, Zhang X, Ren S, Sun J. Deep Residual Learning for Image Recognition. IEEE Conference on Computer Vision and Pattern Recognition. Las Vegas, USA2016.

[6] Dosovitskiy A, Beyer L, Kolesnikov A, Weissenborn D, Zhai X, Unterthiner T, et al. An Image is Worth 16x16 Words: Transformers for Image Recognition at Scale. International Conference on Learning Representations 2020.

[7] He K, Fan H, Wu Y, Xie SQ, Girshick R. Momentum contrast for unsupervised visual representation learning. IEEE/CVF Conference on Computer Vision and Pattern Recognition 2020.

[8] Sowrirajan H, Yang J, Ng AY, Rajpurkar P. MoCo-CXR: MoCo Pretraining Improves Representation and Transferability of Chest X-ray Models. Medical Imaging with Deep Learning. Lübeck, Germany2021. p. 727-43.

[9] Caron M, Touvron H, Misra I, Jegou H, Mairal J, Bojanowski P, et al. Emerging properties in self-supervised vision transformers. IEEE/CVF International Conference on Computer Vision 2021.

[10] Selvaraju RR, Cogswell M, Das A, Vedantam R, Parikh D, Batra D. Grad-CAM: Visual Explanations from Deep Networks via Gradient-based Localization. IEEE International Conference on Computer Vision. Venice, Italy 2017.
